# Emotion Attribution to a Non-Humanoid Robot in Different Social Situations

**DOI:** 10.1371/journal.pone.0114207

**Published:** 2014-12-31

**Authors:** Gabriella Lakatos, Márta Gácsi, Veronika Konok, Ildikó Brúder, Boróka Bereczky, Péter Korondi, Ádám Miklósi

**Affiliations:** 1 Hungarian Academy of Sciences – Eötvös Loránd University, Comparative Ethology Research Group, Pázmány Péter sétány 1/C, Budapest, 1117, Hungary; 2 Department of Ethology, Eötvös Loránd University, Pázmány Péter sétány 1/C, Budapest, 1117, Hungary; 3 Department of Mechatronics, Optics and Information Engineering, University of Technology and Economics, Bertalan Lajos utca 4–6, Budapest, 1111, Hungary; 4 MTA-BME Control Research Group, Bertalan Lajos utca 4–6, Budapest, 1111, Hungary; University of Udine, Italy

## Abstract

In the last few years there was an increasing interest in building companion robots that interact in a socially acceptable way with humans. In order to interact in a meaningful way a robot has to convey intentionality and emotions of some sort in order to increase believability. We suggest that human-robot interaction should be considered as a specific form of inter-specific interaction and that human–animal interaction can provide a useful biological model for designing social robots. Dogs can provide a promising biological model since during the domestication process dogs were able to adapt to the human environment and to participate in complex social interactions. In this observational study we propose to design emotionally expressive behaviour of robots using the behaviour of dogs as inspiration and to test these dog-inspired robots with humans in inter-specific context. In two experiments (wizard-of-oz scenarios) we examined humans' ability to recognize two basic and a secondary emotion expressed by a robot. In Experiment 1 we provided our companion robot with two kinds of emotional behaviour (“happiness” and “fear”), and studied whether people attribute the appropriate emotion to the robot, and interact with it accordingly. In Experiment 2 we investigated whether participants tend to attribute guilty behaviour to a robot in a relevant context by examining whether relying on the robot's greeting behaviour human participants can detect if the robot transgressed a predetermined rule. Results of Experiment 1 showed that people readily attribute emotions to a social robot and interact with it in accordance with the expressed emotional behaviour. Results of Experiment 2 showed that people are able to recognize if the robot transgressed on the basis of its greeting behaviour. In summary, our findings showed that dog-inspired behaviour is a suitable medium for making people attribute emotional states to a non-humanoid robot.

## Introduction

A general requirement for social robots is that they should be able to participate in different interactions with humans. In order to interact socially with humans the robot has to convey intentionality, that is, the human has to think that the robot has goals, beliefs and desires [1]. There is evidence that humans are willing to interpret lifeless objects as social beings, attributing aims, desires, inner states and even personality to them (e.g. [2], [3], [4]). Designers of artificial agents try to exploit this anthropomorphizing tendency and supply social robots with social cues that induce the concepts of intentions in people.

Many scientists in social robotics agree that the main requirements of a complex social interaction include communication, the recognition and expression of emotions, and some rudimentary form of personality [5], [6], [7]. These features are widely thought to increase the believability of artificial agents (e.g. [6], [7], [8], [9]) and enhance the long-term engagement of people toward artificial companions.

The importance of the representation of emotions in artificial agents (or virtual characters) has been recognized long ago in art. According to Thomas and Johnston [10], two of the core animators of the Disney's productions, “it has been the portrayal of emotions that has given the Disney characters the illusion of life”. Many robots and virtual agents (e.g. Kismet, Yuppy, Max, Greta, Probo, EDDIE, Feelix [9], [11], [12], [13], [14], [15], [16], [17]) have been supplied with affective expressions so far in order to examine the contribution of emotions to livingness or to observe humans' perception of the expressive behaviours of robots. Although, it is important to note that most of these studies used only facial expressions to express robotic emotions and questionnaires to analyse the recognition rate of the different emotions. Direct human-robot interactions analysing humans' reactions also on the behavioural level are relatively rare. For example, one of these studies showed that subjects tended to feel less lonely and found the agent (Max) more life-like if it expressed empathy toward them compared to situations, in which the robot did not show emotions or tended to be self-centred [5]. Additionally, the electromyography showed that subjects had higher activity of the masseter muscle (which is one of the muscles of mastication and an indicator of negative valence) when the agent expressed negative empathy (“Schadenfreude”) compared to positive empathy [5].

Many of the present day social robots are built to have humanoid embodiments and their behaviour is designed on the basis of human psychological models. Designers of such humanoid social robots aim to implement human-like behavioural and cognitive features in the robots along with human-like social competencies using human-human interactions as a model (e.g. developing social relationship with humans, gesturing, using speech-based communication etc.) [6], [18]. In the last few years impressive improvements have been achieved in this field and it has been proved by separate studies that humans can successfully recognize emotions displayed by a humanoid face of robots or virtual agents (see also the above mentioned studies and for other examples e.g. [19], [20]; or for a review see [21]). Moreover, some studies provided evidence suggesting that emotions expressed by a humanoid robot face evoke similar emotions in humans as well (e.g. [22], for a review see [21]). Although, a recent study of Chaminade et al. [23] showed that on the level of neural responses humans react differently to the emotional expressions of a humanoid robot and of a human. Besides, again, we have to note that most of these studies have been restricted to the use of facial expressions (instead of using multimodal emotional expressions), which on the other hand requires complex technology both considering the perception and the signalling [24].

In fact, human-human interactions are very complex since they are generally symmetric, develop since birth and are based on the use of language. Hereby, it is extremely hard for robot designers to mimic human behaviour successfully and the robots mimicking human behaviour will never be perfect “humans”. This can lead to mismatches between the appearance and the behaviour, which means that the users' prior - often unrealistic – expectations, mostly based on the appearance, will be violated resulting in a feeling of unease [21], [24], [25]. This is the well-known phenomenon of the “uncanny valley” [26], that is, agents which are very but not totally similar to humans, induce aversion in people.

Other companion robots are designed to have rather pet-like appearance (e.g. PLEO, AIBO, PARO [27], [28], [29]) and have also been used as alternatives to animal assisted therapy [29], [30]. However, the behavioural repertoire of these pet-like social robots is very limited and for this reason, compared to animal pets they proved to be less successful in maintaining humans' interest in long term [31], [32].

Hereby, we suggest an alternative approach, that is, the application of animal models for the behavioural design could provide an important alternative in the development of future social robots [18]. Human-animal interaction can provide a useful alternative since similarly to human-robot interactions human-animal interactions are asymmetric, they are much simpler than human-human interactions, they may start at any age and importantly, human-animal social interactions develop without using language from the part of the animal via non-verbal communicational behaviour. We argue that social robots should not necessarily be human-like -nor pet-like in appearance -but rather functional with regard to their roles in the human community, and their social behaviour should mirror this function as well, taking into account the embodiment and the technological constraints [18].

In line with this argument, we suggest that instead of using complex human behavioural models the social behaviour of companion robots could be based on the abstractions of the behaviour observed in companion animals when they interact with humans.

Accordingly, human-robot interaction could be regarded as a specific form of inter-specific social interaction. This situation has its natural analogies in human-animal interaction, including humans' specific relationship with domesticated animals (such as dogs, cats, etc., see below) or wild living species, like dolphins [33]. The analysis of these analogies (human-animal interactions) enables us to build behaviour models on inter-specific interactions based on naturalistic evidence.

Domestic animals seem to be the best candidates for providing the inspiration to design robot behaviour because they are able to develop effective social interaction with humans [18]. Human-dog interaction has already been suggested as a framework to model human-robot interactions before [18], [34] (although others argue with its effectiveness [35]). Indeed, dogs excel with their social skills from domestic animals [36]. The dog was the first species that joined the human society [37] and during the domestication process dogs successfully adapted to the human social environment (e.g. [38]) and developed specific inter-specific communication skills with humans on a daily basis [36]. Dogs engage successfully in complex social interactions (e.g. cooperation) with humans despite their less complex cognitive capacities. Furthermore, dogs' behaviour is very well-documented, including catalogised description of their behaviour [39], [40]. Researchers found that attachment between dogs and owners is functionally very similar to that of infants and their parents [41], [42], [43]. The ability of dogs to develop such a strong bond with a human can play a key role in their long-term social relationships [36]. We also know that owners can recognize the behaviour of dogs, which corresponds to its emotional states, for example, people are skilful at identifying the emotional content of barks [44]. There is much to be learnt from dogs about how they achieve a relatively complex level of social interaction with humans [18], hence we assume that dogs provide an appropriate animal model for building social robots.

Note, however, that when we propose to design emotionally expressive behaviour of robots using the behaviour of dogs as inspiration, we do not want to copy dog behaviour in any direct sense. Our goal is to observe the behavioural organisation of various behaviour systems in dogs and build a functional analogue into our robots using multimodal channels. Then these dog-inspired robots can be tested during direct interactions with humans in order to see how they can manage social interactions in an inter-specific context.

Here we present the results of two experiments in which we examined humans' ability to recognize two basic and a secondary emotion expressed by a robot. Apart from getting a subjective feedback from the subjects, we were interested in the behaviour of the participants during the interactions with the robot. We also examined whether interacting with the robot can change the subjects' attitude towards robots in general, and whether there is any gender effect in human-robot interactions.

## General Method

### The robot

**Figure 1:**
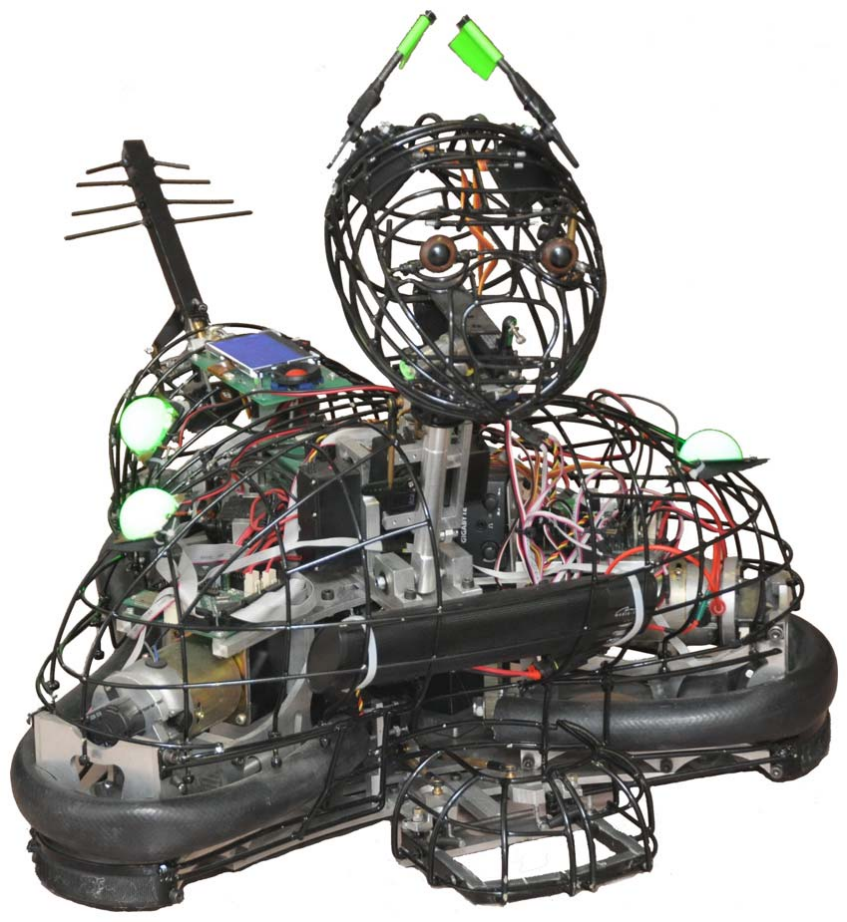
Photo of the robot, called “MogiRobi”, that we used in the present study.

The robot (MogiRobi) (Fig. 1) used in this study was built by Balázs Varga, Bence Kovács, and Géza Szayer from the Department of Mechatronics, Optics, and Mechanical Engineering Informatics at the Budapest University of Technology and Economics in collaboration with the Department of Ethology at Eötvös Loránd University [45]. This robot is controlled by the means of a remote controller via bluetooth connection. It travels freely around in the room and moves its head, rear-antenna and ear-like appendices independently from the body. The two ‘ear-like appendices’ could be rotated either upward or backward, and a rear antenna could also be positioned upward or downward, and moved sideways (3 degree of freedom (DOF) head, 2 DOF neck, 1 DOF ear-like appendix, 2 DOF rear antenna).

### Ethics statement

The work was conducted according to relevant national and international guidelines. A written informed consent was signed by all participants, however the data was analysed anonymously. In addition, we consulted with the institutional review board (Institutional Review Board and Ethics Committee of the Institute of Biology, Eötvös Loránd University). They provided a written ethics approval for the experiments and a written statement that there is no need for the approval of higher ethics committees.

The individual in this manuscript has given written informed consent (as outlined in PLOS consent form) to publish these details and images.

### Procedure

After having signed the informed consent, human subjects filled out three pre-test questionnaires (S1 Appendix) at their computer at home or at the department before they met the robot. It took approximately 15 minutes to complete the questionnaires. Then they participated in two experiments, which followed each other in a fixed order: (1) Emotion Attribution, (2) Guilt Attribution. (A third test, Personality Attribution was also performed later, but the results of this test will be reported elsewhere and will not be discussed here further). The experiments took place at the Family Dog Project laboratory, Eötvös Loránd University (Budapest, Hungary), in a testing room (4.6 m×3.8 m), which had 3 doors on 3 different walls. The room was equipped with 4 cameras (one camera on the top of each wall).

The experiments lasted approximately 20 minutes. After the tests the subjects filled out two post-test questionnaires (S2 Appendix) what took about 10–15 minutes to complete.

The pre- and post-test questionnaires contained items regarding the subjects' demographic data, a technological attitude scale, a Negative Attitudes towards Robots Scale (NARS) [46], and items about the robot's livingness and emotions (see S1 and S2 Appendices). The questionnaires will be further described in more details at the end of Experiment 2 where we will present also the analyses of this data.

The description of the behaviour tests will follow in separate sections (Experiment 1 and Experiment 2, see below). During the tests the robot was always controlled from an adjoining room by an experimenter (who could follow the actions of the robot on a computer monitor), but the participants were unaware of this condition.

Before the experiments, the subjects were informed that they were supposed to interact with a robot and we explained them what to do during the test. Before Experiment 1 the participants did not know anything about how the robot looks like and what it is able to do. We only answered participants' questions about the procedure of the behaviour tests but did not answer questions regarding the robot's skills or behaviour. In case of such questions we asked the participants to wait for the answers until the end of the behaviour tests.

### Data collection and analysis

Both experiments were video-recorded for later analysis. Solomon Coder beta 10.11.29 (Copyright © 2010 András Péter; http://solomoncoder.com) was used for behavioural coding.

SPSS for Windows was used for all statistical analyses. Using the Shapiro-Wilk test, we found that most of the behavioural variables and questionnaire scales were not normally distributed, so we used non-parametric tests.

We used Mann-Whitney tests to investigate the effect of independent variables of the demographic questionnaire on other questionnaire and behavioural variables. We used Wilcoxon matched pairs test to compare repeated measures and order effects. Binomial tests were used to analyse whether subjects' choices differed from chance level, and X^2^/Fisher-exact tests were used to observe whether ratios differ among groups.

Corrections for multiple comparisons were calculated in MATLAB.

## Experiment 1: Emotion Attribution in a Play Situation

### Introduction

It is already well known that we can recognize human emotions with a generally high level of accuracy also in case of people from different cultural/ethnic groups [47]. Moreoever, separate studies have demonstrated that humans attribute a wide range of emotions also to dogs (e.g. [48]), and it can be assumed that this ability of attributing emotions to dogs is not strongly affected by local culture either.

Bates et al. [8] claimed that emotional behaviour makes social robots more believable and attractive for humans. Bruce and colleagues [49] found that if they supplied their robot with a face that expressed adequate emotions, people were more willing to interact with it, that is, they more frequently stopped in their way to answer some questions the robot asked from them. Leite and colleagues [50] reported that users understood better the actual state of a chess game (whether they were losing or winning) if the iCat robot opponent produced the appropriate emotional expressions. Thus, it seems that the robot's expression of emotions facilitates the human-robot interaction [6] and may contribute to the long-term engagement of humans towards artificial companions.

In the present observational study we investigated the effects of the emotionally expressive behaviours of a robot that were inspired by functionally analogous behaviours observed in dogs. By the means of both direct (behavioural observations) and indirect (‘Robot Anthropomorphising Questionnaire’: see below) measures we wanted to study whether the non-human ways of emotion expression influenced human-robot interactions. We provided our companion robot with two kinds of emotional behaviour (joy and fear) designed on the basis of dogs' expressive behaviour (canine ‘happy’ and ‘fearful’ behaviour), and studied whether people recognized and attributed the appropriate emotion to the robot, and interacted with it accordingly.

Recognition of joy and fear has been examined in many different robotic platforms so far [14], [15], [16], [17], which gives us the opportunity to compare the effectiveness of emotionally expressive behaviours inspired by different animal- and human-like models. In addition, the expression of “joy” and “fear” is especially important in robotic companions as it can significantly improve the quality of the human-robot interaction and contribute to the user's long-term engagement toward the robot. In special contexts these emotions are related to attachment in humans and animals (stress in separation from important others and joy at reunion with them). These attachment-related emotions are assumed to help in maintaining social bonds (e.g. [51]).

Previous studies showed that fear is generally more difficult to recognize for people [14] and that animal-like features have a positive effect on robotic emotion recognition [16]. However, these studies used mostly facial expressions to express emotions and no direct behavioural observations were taken to analyse humans' reactions. In this regard the present experiment provides two important novelties.

We hypothesized that the dog-inspired expressive behaviour will be readable for humans and they will attribute the appropriate emotions to the robot. In addition, in line with the results of earlier studies we expected a lower emotion recognition rate in case of the ‘fearful’ emotional behaviour.

### Method

#### Subjects

71 individuals: 37 men and 34 women between the ages of 19 and 34 years (M = 24.39, SD = 3.97) participated in the Emotion Attribution Test, of which the data of 48 individuals was analysed (26 men and 22 women between the ages of 19 and 34 years (M = 24.5, SD = 3.86)). The remaining 23 individuals were excluded from the further analysis due to technical problems (some parts of the robot did not function properly) or due to mistakes in the procedure.

#### Experimental design

The experiment was conducted in a wizard-of-oz scenario (the participants interact with a computer system that they believe to be autonomous, but which is actually being operated by an unseen human being). For the testing we used two different-coloured balls with which MogiRobi and the subject interacted. MogiRobi's reaction toward the balls was one of two different kinds: preference vs. non-preference (see detailed description below).

There were two independent variables: the robots' reaction to the ball, that is, preference vs. non-preference, and the colour of the ball, that is, yellow vs. black-and-white (see below). We used a within-subject design: each subject participated in both conditions (preference and non-preference), but the order of these two conditions and the colour of the preferred ball were counterbalanced among subjects. Twenty-four subjects participated in the ‘preferred ball’ condition first, and in the ‘non-preferred ball’ condition second; out of the 24 participants in case of 12 participants the preferred ball was the yellow one, while in the case of the other 12 participants the preferred ball was the black-and-white one. Another 24 subjects participated in the ‘non-preferred ball’ condition first, and in the ‘preferred ball’ condition second; again, for 12 of these participants the preferred ball was the yellow one, while for the other 12 participants the preferred ball was the black-and-white one.

#### Procedure

**Figure 2:**
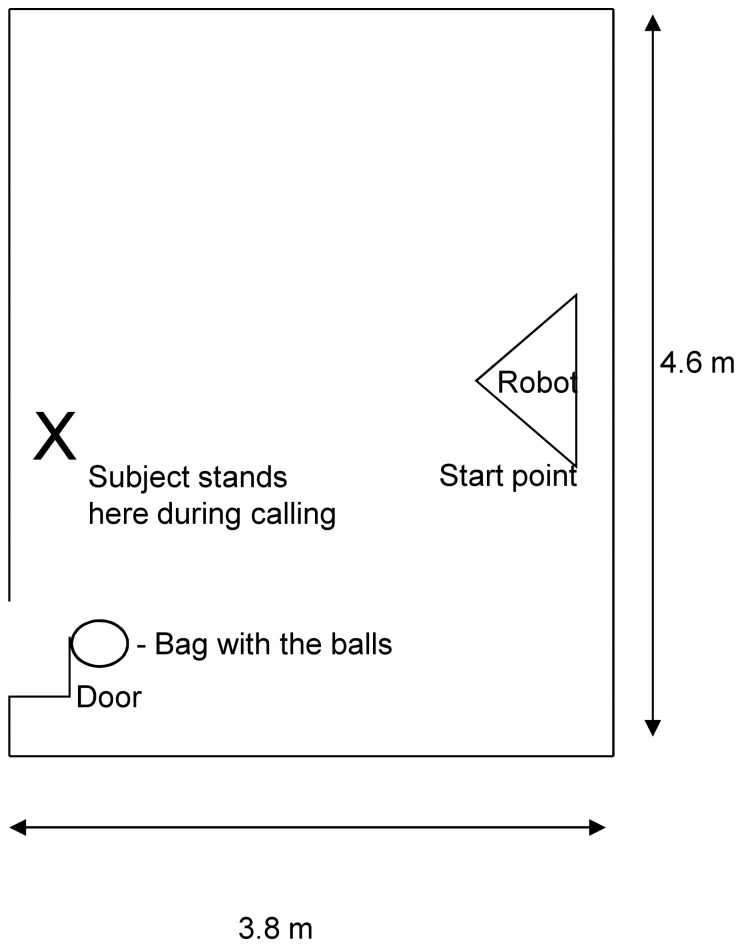
Experimental layout of the Emotion Attribution Test (Experiment 1).

The two balls (a yellow tennis ball and a black-and-white ball of the same size) were placed in a bag at the beginning of the test. The bag (30×40 cm), in which the balls could be placed during the testing, was fixed on the handle of one of the doors. MogiRobi was placed in the testing room at the predetermined position (see Fig. 2).

The experiment consisted of four episodes.

*Greeting* (15–20 s): The robot's starting position was in the back of the room, it stood with the ear-like appendices upward, holding his steady antenna in upright position. The behaviour of MogiRobi was modelled on the greeting behaviour displayed by companion dogs. We asked the subjects to enter the room and call MogiRobi. The robot was turning its head toward the entering person, moved its antenna sideway mimicking the tail wagging of a happy dog and started to move its antenna. When called by the subject, the robot approached her/him wagging its antenna. Arriving at the subject, MogiRobi lowered its antenna and ear-like appendices.

*Directed play 1, 2* (2×1 minute): The subject was told to play with MogiRobi for 1 minute with each ball. There were two conditions. In one condition the robot was attracted to one of balls (‘preferred ball’ condition), while in the other condition it showed avoidance toward the other ball (‘non-preferred ball’) (see below the details of the expressed behaviour). The order of these two conditions and the colour of the preferred ball were counterbalanced among subjects (see the experimental design). The not used ball was always put away in the bag. Subjects were naive, that is, they had no a priori knowledge about the preferences of the robot.

*Free play* (1 minute): The two directed play episodes was followed by a free play episode without break. The subject could play again with MogiRobi, but now he/she was free to play with either of the balls. He/she was allowed also to switch the balls, but only one ball could be used at a time, the other ball had to be kept in the bag. During the free play MogiRobi expressed the corresponding emotions related to the specific ball.

The experimenter indicated the end of each episode by knocking on the door.

#### Emotional behaviour of the robot

**Figure 3:**
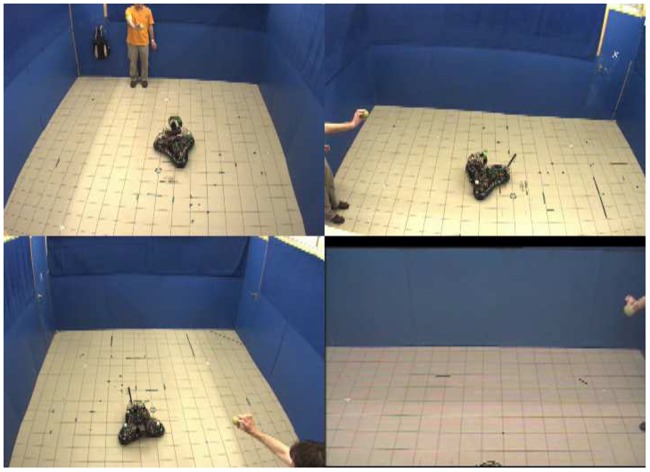
Photo of the ‘Preferred ball’ condition in Experiment 1.

When the participant played with the preferred ball (in the Preferred ball condition, and during the Free play episode) the robot expressed behavioural features that resembled the canine ‘happy’ behaviour: upward tail and ear-posture, tail-wagging, approaching the targeted object [40]; [52]. When the subject played with the non-preferred ball (in the Non-preferred ball condition, and during the free play episode) the canine behaviours typical in frightening situations (pulled-back tail and ears, holding maximum distance from the object of fear) were applied. Note, however, that the robot's ear-like appendices and antenna, showed relatively little physical resemblance to the ears and tail of dogs (see Fig. 1). Based on the above characteristics the robot's expressive behaviour was designed as follows:

Preferred ball condition: When the subject took out the ball, the robot lifted its antenna and ear-like appendices, and wagged its antenna. When the subject threw/rolled the ball, the robot always approached it and brought it back to the subject (Fig. 3).

**Figure 4:**
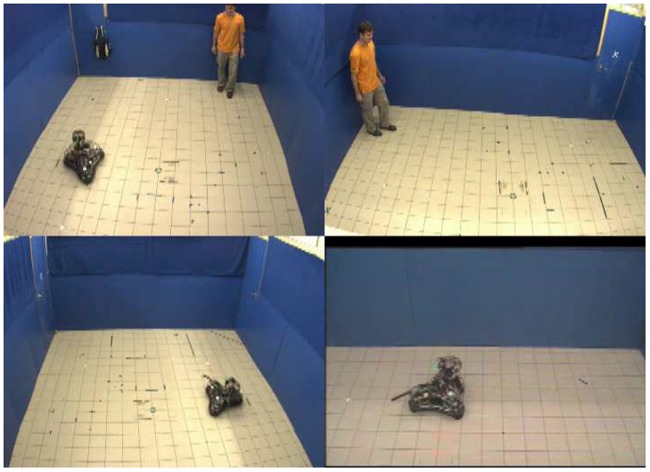
Photo of the ‘Non-preferred ball’ condition in Experiment 1.

Non-preferred ball condition: When the human took out the ball from the bag, the robot stopped moving the antenna, went closer to the ball, oriented its head towards it and suddenly lowered down its antenna and ear-like appendices, and backed. It tried to maintain as large distance from the ball as possible. When the human subject threw/rolled the ball toward the robot, it moved to the other side of the room (Fig. 4).

#### Dependent variables

As dependent variables we analysed the items of the questionnaire that the subjects filled out after the behavioural test (‘Robot Anthropomorphising Questionnaire’, see below), we analysed the subjects' verbal and nonverbal behaviours during the whole behavioural test (in all four episodes), and the time percentage the subjects played with each ball in the Free play episode.

#### ‘Robot Anthropomorphising Questionnaire’ (After the behavioural observation in Experiment 1)

The robot anthropomorphising questionnaire (S3 Appendix) was a post-test questionnaire after the behavioural observation in Experiment 1, which contained open ended and forced choice questions about the participants’ view about the robot's behaviour and inner states. We were interested whether the participants recognized the difference in the robot's behaviour toward the two balls, how they interpreted the different behaviours, and whether they used spontaneous emotion (or inner state) attribution for the interpretation. Besides, we wanted to find out which of Ekman's six basic emotions (anger, fear, happiness, surprise, disgust, sadness; [53]) the subjects felt they had experienced during the interaction with the robot. It was also our aim to reveal on what behaviour elements of the robot the participants based their emotion attributions.

We expected that the participants would recognize the difference in the robot's behaviour toward to two balls and that they would attribute inner states to the robot. In addition, we expected that participants would be more successful in recognizing the robot's happy behaviour compared to the fearful one.

#### Behaviour coding

We recorded the time percentage the participants played with each of the balls in the Free play episode and coded the participants' verbal and nonverbal behaviours (similarly to [46], [54], [55], [56]) during the whole behavioural test (in all four episodes). Behaviour variables were chosen inductively based on material after watching all the experimental videos. The specific behaviour elements coded are presented in Table 1. We counted the frequencies of the coded behaviour elements, and formed scales from those elements that had similar function or indicated similar inner states of the participant (we had no pre-conceptual categories, the scales were formed inductively). Values of the scales are the sum of the corresponding variables (e.g. total frequency of any positive emotional display). These scales are the following (the composition of the scales can be seen in Table 1):

**Table 1:** The coded behavioural variables in the Emotion Attribution Test, and the association with the four scales used in the analysis.

Variable	Definition	Communicative channel	Name of the scale
Calling by name	Naming the robot, like “Mogi”, “Robi”, “MogiRobi” or using nicknames like “buddy”.	verbal	CommandAtt
Calling in	Encouraging the robot to go to the subjects, like “Come!”, “Come here!”, “Would you come here?”.	verbal	CommandAtt
Attention getting	Calling the attention of the robot verbally, like “Look!”, “Listen!”, “Hey!”, or with voices, e.g. by whistling.	verbal/acoustic	CommandAtt
Command („fetch!”)	Giving verbal commands to the robot concerning the fetching of the ball, like “Fetch it!”, “Go for it!”, “Catch it!”.	verbal	CommandAtt
Pointing	Stretching one arm with extended index finger in the direction of the target (usually, the ball).	non-verbal	CommandAtt
Showing of the ball	Holding the ball in hand and bringing it closer to the robot, holding it in front of the robot (in the scope of the robot).	non-verbal	CommandAtt
Asking about the rules	Asking information from the experimenter about rules or permitted/forbidden/expected behaviour of the subject, e.g. “How should I throw the ball?”, “May I move?”, “Can I touch him?”.	verbal	Confuse
Asking about the robot	Asking information from the experimenter about the skills, abilities or features of the robot, e.g. “Does he recognize the ball?”, “What kind of commands does he know?”.	verbal	Confuse
Expressing incomprehension	Expressing incomprehension, embarrassment or confusion, like “What's up?”, “What's now?”, “What's the problem?”.	verbal	Confuse
Sad voice (without word)	Expressing sad, disappointed feelings with voice only, without words (“Ohh” with a descending intonation).	acoustic	NegEmo
Discouraging	Discouraging, frowning or disapproving the robot's behaviour or expressing dissatisfaction, resentment, displease, e.g. “Tut!”, “Hey, you know this!”, “Be a little bit more interactive!”, etc.	verbal	NegEmo
Hand on hip	Putting the hands (or at least one hand) on the subject's hip.	non-verbal	NegEmo
Arms outspread	Spreading out the arms (or at least one arm), that is, lifting them sidelong and straight.	non-verbal	NegEmo
Any other gesture/motion that expresses negative emotion	Any other gesture/motion that expresses negative emotion (e.g. shrug, scratching of the chin, spatting/waving/flicking with the hands resignedly)	non-verbal	NegEmo
Praising	Praising the robot, like “Well done!”, “You are clever!”, “Good!”.	verbal	PosEmo
Saying thanks	Saying thanks to the robot, e.g. “Thank you”, “Thanks”.	verbal	PosEmo
Expressing liking	Expressing general liking, satisfaction or positive feelings with the robot or with the experiment, e.g. “Cool!”, “This is great!”, “It's cute!”, “It's funny!”.	verbal	PosEmo

Positive emotional behaviours (PosEmo): Sum of behaviour frequencies indicating positive emotion.

Negative emotional behaviours (NegEmo): behaviours indicating negative emotion.

Confusion related behaviours (Confuse): behaviours indicating confusion, embarrassment or indecisiveness.

Commands and attention-calling behaviours (CommandAtt): behaviours functioning as commands, instructions or attention getters.

Twenty percent (N = 10) of the videos were coded also by a second observer. Inter-observer reliability was determined for each variable category by counting Cohen's Kappa coefficients between the coding of the two observers. The reliability can be considered excellent, Cohen's Kappa coefficients ranged from 0.93 to 1.

#### Coding of the open-ended questions

The answers to the open-ended questions of the robot anthropomorphising questionnaire (e.g. “Why did you play more with that ball?” or “What was the difference?”) were categorized after content-analysis. Categories were developed inductively, based on the data. We assigned the answers to one or more of the following five categories: emotions, behaviour, cognition, expressiveness, and other. These categories and their definitions are provided in Table 2. One answer could be assigned to more categories (e.g. if it contained reference to both emotion and cognition), but the category “behaviour” was exclusive to any other category.

**Table 2:** Questionnaire categories used in the Robot anthropomorphising questionnaire.

Category	Definition	Example
Emotions	The subject explicitly refers to some emotion of the robot.	“The robot was afraid of the ball.”
Behaviour	The subject refers only to the observable behaviour of the robot.	“The robot did not retrieve the ball.”
Cognition	The subject refers to the cognition or perception of the robot.	“The robot did not recognize the ball.”
Expressiveness	The subject refers to some emotional expressive behaviour of the robot (without mentioning specific emotion).	“The robot escaped from the ball.”
Other	The subject refers to something else.	“I did not like that ball.”

### Results

First, we checked whether the subjects (N = 48) reacted differently to the appearance of the balls independently from the condition, in which they were used. By analysing behaviour data, we found that there was no significant difference in the time spent with playing with the two balls in the free play episode (W = 354; p = 0.61). When we analysed the answers of the questionnaire item “Which ball did you play more with?” 23 subjects reported that they played more with the black-and-white ball and 25 with the yellow. Thus for further analysis we merged the data concerning the two balls.

**Figure 5:**
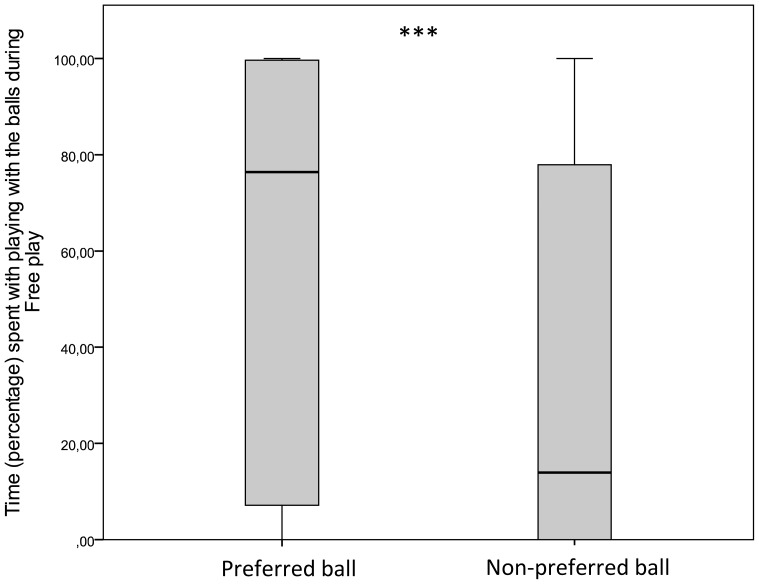
Time percentage spent playing with the ‘preferred’ and the ‘non-preferred’ ball during the Free play episode.

Next, we found that participants spent more time with playing with the ‘preferred’ ball compared to the ‘non-preferred’ one in the *Free play* episode (W = 828; p<0.001, Fig. 5) and this was also supported by questionnaire data: participants reported that they played more with the with the ball, which in that condition was the ‘preferred’ ball compared to the ‘non-preferred’ ball (Χ^2^ = 24.125; p<0.001) (see also Table 3).

**Table 3:** Descriptives and results for the behaviour variables in the Emotion Attribution Test.

Behavior variable	Median	Interquartile range	W	p
Time spent with playing with the yellow ball in the Free Play episode (in time percent)	76.42	96.105	354	0.61
Time spent with playing with the black-and-white ball in the Free Play episode (in time percent)	13.935	79.0575		
Time spent with playing with the “preferred” ball in the Free Play episode (in time percent)	97.24	28.775	828	<0.001
Time spent with playing with the “non-preferred” ball in the Free Play episode (in time percent)	0	25.14		
Sum of behaviour frequencies indicating positive emotion (“PosEmo” scale) in Directed Play 1.	0	0	−105	0.09
Sum of behaviour frequencies indicating positive emotion (“PosEmo” scale) in Directed Play 2.	0	0.75		
Sum of behaviour frequencies indicating negative emotion (“NegEmo” scale) in Directed Play 1.	0	1	−6	0.92
Sum of behaviour frequencies indicating negative emotion (“NegEmo” scale) in Directed Play 2.	0	1		
Sum of behaviour frequencies indicating confusion (“Confuse” scale) in Directed Play 1.	0	1	167	0.03
Sum of behaviour frequencies indicating confusion (“Confuse” scale) in Directed Play 2.	0	0		
Sum of behaviour frequencies functioning as commands or attention getters (“CommandAtt” scale) in Directed Play 1.	3	4	164	0.19
Sum of behaviour frequencies functioning as commands or attention getters (“CommandAtt” scale) in Directed Play 2.	2	3.75		
Sum of behaviour frequencies indicating positive emotion (“PosEmo” scale) in the “preferred ball” condition	0	1	240	<0.001
Sum of behaviour frequencies indicating positive emotion (“PosEmo” scale) in the “non-preferred ball” condition	0	0		
Sum of behaviour frequencies indicating negative emotion (“NegEmo” scale) in the “preferred ball” condition	0	0	−198	<0.001
Sum of behaviour frequencies indicating negative emotion (“NegEmo” scale) in the “non-preferred ball” condition	0.5	2		
Sum of behaviour frequencies indicating confusion (“Confuse” scale) in the “preferred ball” condition	0	0	−179	0.022
Sum of behaviour frequencies indicating confusion (“Confuse” scale) in the “non-preferred ball” condition	0	1		
Sum of behaviour frequencies functioning as commands or attention getters (“CommandAtt” scale) in the “preferred ball” condition	2	3	−452	<0.001
Sum of behaviour frequencies functioning as commands or attention getters (“CommandAtt” scale) in the “non-preferred ball” condition	3	7.5		

When subjects had to explain why they played more with one of the balls, they referred to the above-mentioned categories in the following ratios: emotions (35.4%), behaviour (25%), cognition (20.8%), expressiveness (16.7%), and other (18.8%).

An overwhelming majority (95.8%) of the subjects said that the robot reacted differently to the two balls. When we asked what the difference was in the robot's reaction, 43.8% of them referred to emotions, 29.2% to cognitions, 22.9% to expressive behaviour, 16.7% to general behaviour, and 2.1% to other.

The median response was 4 (from the maximum 5) to the question “How much did it seem in this test that MogiRobi possessed emotions?”. Happiness, fear and interest were the main emotions subjects reported spontaneously to open-ended questions (Table 4). Happiness and interest were the most frequently mentioned emotions with regard to the ‘preferred ball’, and fear in the case of the ‘non-preferred ball’.

**Table 4:** Ratio of subjects (in percent) who reported the given emotions and expressions spontaneously (open ended), regarding overall emotional expressions of MogiRobi (question 7) and the robot's emotions specifically toward the two balls (questions 8 and 9).

	Overall emotions	Emotions toward the “preferred” ball	Emotions toward the “non-preferred” ball
Happiness	**59.2**	**47.9**	4.2
Fear	**26.5**	0	**33.3**
Interest	**24.5**	**33.3**	2.1
Playfulness	**14.3**	**18.8**	0
Excitement	**10.2**	**12.5**	2.1
Enthusiasm	4.1	4.2	0
Sadness	8.2	0	4.2
Indifference/neutral	6.1	2.1	**35.4**
Attention	6.1	8.3	6.3
Dislike/Rejection	6.1	4.2	**12.5**
Affection/Love	0	**12.5**	0
Other	6.1	6.1	**14.6**
Doesn't know/Irrelevant answer	8.2	0	6.3

For better comprehension, percentages over 10 are indicated in bold. Note that one subject could indicate more than one emotion.

**Figure 6:**
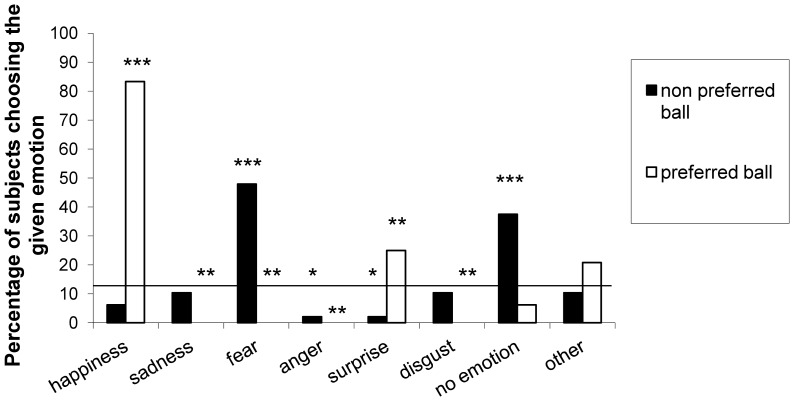
Percentage of subjects choosing the given emotions in the forced-choice questionnaire in case of both the ‘preferred’ ball and the ‘non-preferred’ ball.

Corresponding results were obtained in response to the forced-choice questions (see S3 Appendix). Binomial tests were carried out to compare subjects' choices to the chance level (as there were 8 options- 6 emotions, 1 “no emotion” and 1 “other”, the chance level was determined as 0.125). In case of the ‘preferred ball’, the choices differed significantly from chance level except for the choice of “no emotion” and “other”. Subjects chose happiness and surprise more frequently than chance (p<0.001 and p<0.01, respectively), and chose all the other emotions less frequently than chance (p<0.01 in all cases). In case of the ‘non-preferred ball’, percentage of choices of happiness, sadness, disgust and “other” did not differ from chance, but subjects chose fear and “no emotion” more frequently than chance (p<0.001) and anger and surprise less frequently than chance (p<0.05) (Fig. 6).

**Figure 7:**
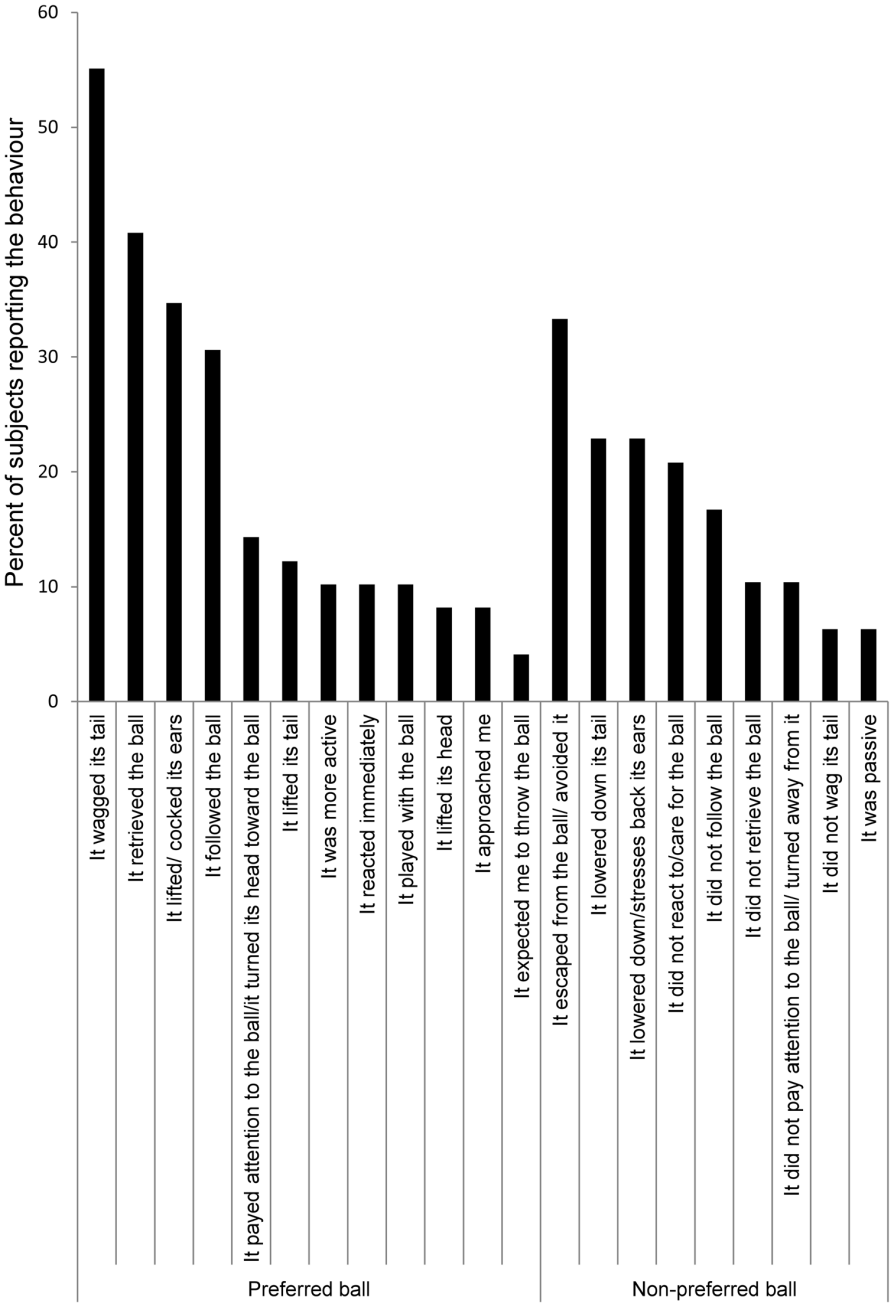
Percentage of subjects reporting the given behaviours when describing on what behaviours they based their emotion-attribution in case of the ‘preferred’ and the ‘non-preferred’ ball.

Subjects reported that they based their emotion-attributions on specific behaviours of the robot both in the case of the ‘preferred’ and the ‘non-preferred’ ball. They referred to specific actions, e.g. following and retrieving the ball, lifting its ear-like appendices and antenna or being passive (see Fig. 7 for a more detailed description).

The comparison of the subjects' behaviour in the two conditions revealed that when the robot displayed ‘happy’ behaviour, subjects also expressed more positive emotions (“PosEmo” scale) (W = 240; p<0.001), less negative emotions (“NegEmo” scale) (W = −198; p<0.001), less confusion/embarrassment (“Confuse” scale) (W = −179.0; p = 0.022) and their commanding and attention-getting behaviour was also less frequent (“CommandAtt” scale) (W = −452; p<0.001) than when the robot behaved in a ‘fearful’ way (see also Table 3).

We checked also whether the order of the two conditions had an effect on the subjects' behaviour. We found a significant effect of order only in the case of the confusion related behaviours, showing that subjects showed more confusion in the first Directed Play episode (independently from whether it was a “preferred ball” or a “non-preferred ball” condition) than in the second Directed Play episode (“PosEmo”: W = −105, p = 0.09; “NegEmo”: W = −6, p = 0.92; “Confuse”: W = 167, p = 0.03; “CommandAtt”: W = 164, p = 0.19) (see also Table 3).

## Experiment 2: Guilt Attribution Test

### Introduction

A recent study has demonstrated that robots which make mistakes and show partly incongruent multimodal behaviour are perceived as more likable by the users, suggesting that small mistakes enhance the perceived lifelikeness, likability and believability of robots [57].

Along these lines one can also assume that robots that show guilty behaviour when making mistakes would be perceived more life-like and believable as well, but it has never been examined so far if people can attribute guilt (a secondary emotion) to a robot.

From previous studies we know that dog owners ascribe guilt to dogs [48]. Horowitz [58] supposed that the ‘guilty look’ of dogs is only a response to the owner's behaviour, and its function is to avoid punishment. In another study owners successfully determined whether or not their dogs performed a disallowed action [59]. Analysing individual dogs' behaviour, Hecht et al. [59] identified specific changes in the greeting behaviour of dogs during the experiment. These specific changes in the dogs' behaviour helped the owners to recognize whether or not the dog did the misdeed. Owners reported that dogs who have performed the disallowed action in their absence displayed guilt-associated behaviours towards them. Taken together, we can assume that behaviours associated with guilt exist in the dog and some owners even tend to attribute the feeling of human-like guilt to dogs. In this study, however, we were not interested in investigating the mental representations beyond behaviours that can be connected to emotions in dogs, because the viewers' subjective impressions were in the focus of our observations.

In Experiment 2 we investigated whether participants tend to attribute guilty behaviour even to a non-living creature, a robot in some relevant context. The robot's behaviour was determined on the basis of the behavioural descriptions of dogs observed in similar situations [58], [59].We investigated whether relying on the robot's greeting behaviour human participants could detect if the robot transgressed a predetermined rule. We hypothesized that the dog-inspired behaviour implemented in the robot will be effective in communicating guilt to the users, hence the participants will be able to detect if the robot transgressed the predetermined rule.

As a further goal of the present study, we aimed to examine how participants teach a robot during human-robot interactions, more closely how they try to get the robot not to do an undesired act. Machine-learning and the potential methods for teaching robots are broadly investigated areas of social robotics. However, only very few studies examined this question directly from the side of the users, more closely, the question how humans spontaneously try to teach a robot. Thomaz and Breazeal [60] studied how people want to teach a Reinforcement Learning agent through human-agent interaction, applying a Java-based simulation platform. Although the results of this study have provided exceptional contributions to the research field of robot-learning by demonstrating the importance of analysing the human-teacher/robot-learner relationship in order to improve the robot's learning behaviour, it also had some limitations. The possible teaching methods were already limited by being based on reinforcement learning and using a game interface instead of a robot for the interactions. In the present study we used the above described robot and we analysed the subjects' teaching methods observed in direct human-robot interactions. The robot was remotely controlled in the experiment and independently of the teaching method that the naïve participants used, it showed learning during the interaction. This let us analyse what teaching methods are the most preferred ones by the users when meeting a robot.

### Method

#### Subjects

In this experiment we tested the same 71 individuals who had participated in Experiment 1 (37 men and 34 women between the ages of 19 and 34 years (M = 24.39, SD = 3.97)). The data of 44 participants could be analysed (26 men and 18 women between the ages of 19 and 34 years (M = 24.16, SD = 3.96)), the remaining 27 individuals were excluded from the further analysis due to technical problems (some parts of the robot did not function properly) or due to mistakes in the procedure (participants did not act according to the instructions).

#### Experimental setup

**Figure 8:**
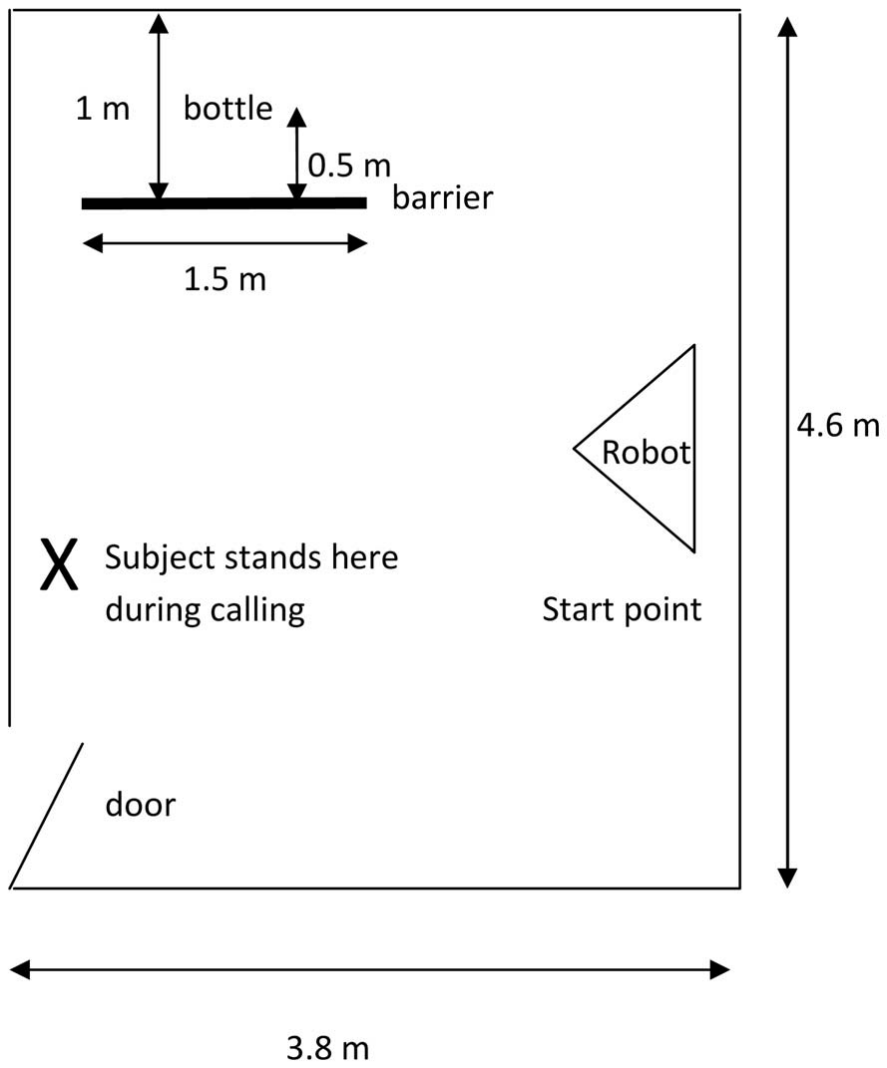
Experimental layout of the Guilt Attribution Test (Experiment 2).

A bottle and a 1.5 m long barrier to prevent the subjects from seeing the bottle was placed near the wall in the testing room (4.6 m×3.8 m). MogiRobi was also placed in the testing room in a predetermined position (Fig. 8).

#### Experimental design

There was one independent variable: the experimental group, that is, the ‘Guilty greeting’ group vs. the ‘Typical greeting’ group (see below). A between-subject design was used: half of the subjects were assigned to the ‘Guilty greeting’ group, while the other half of them were assigned to the Typical greeting' group (each participant participated only in one condition).

#### Procedure

Participants were divided into two experimental groups. In the ‘Guilty greeting’ group (N = 22) the robot showed ‘guilty greeting’ behaviour at the end of the test. In the ‘Typical greeting’ group (N = 22) the robot showed the typical, everyday greeting behaviour. The greeting behaviour of the robot was inspired by the greeting behaviour observed in dogs interacting with humans (see below). Except the greeting behaviour at the end of the test phase the robot's behaviour was the same in the two groups.

The test consisted of four episodes. During the test the robot was controlled from an adjoining room.

Before the test the Experimenter said to the subject that after entering the rooms/he had to wait for a short time and call the robot exactly the same way as they did previously in the Emotional Attribution Test. After the first episode (Greeting I) the experimenter entered the room and explained the further instructions to the subjects.

1. *Greeting I*: The robot was placed in a resting posture in the test room. The subject entered the room, closed the door, stepped aside and waited. At the moment the subject entered, the robot showed attentive behaviour toward him/her by orienting its head and turning the ear-like appendices upwards. Upon being called by the subject the robot approached the subject and stopped in front of him/her, looked up with ‘ear-like appendices’ back and wagged its ‘antenna’ (typical greeting). After the greeting episode the experimenter entered the room.

2. *Teaching I*: Apart from analysing the teaching behaviour of the human subjects, the purpose of this test episode was to pretend that the robot is able to perceive and perform actions on commands given by the participants. We wanted them to believe that the robot is able to learn. The task to be taught was to turn around its own axis on command by the subject. The experimenter explained the task to the subject in about 2 minutes, while the robot was exploring the test room. The subjects were allowed to do anything to get the robot perform the desirable act: they could talk to the robot, use gestures and commands, lure it, and praise it. In these learning situations the same rules were applied in the control of the robot: (1) at the first attempt when the subject tried to show or tell the robot what to do, the robot turned its head to the direction that was indicated by the human; (2) on the next teaching attempt the robot displayed a full turn. (3) Then the experimenter said to the subject to try it again and make the robot to turn for a command or a hand signal to see whether it really learned the action. When the subject did so, as a response the robot showed a perfect turn. This teaching episode was about 5 minutes long.

3. *Teaching II*: The aim of this test episode was twofold: (1) we wanted to observe how the participants try to get the robot not to do the undesired act, and (2) subjects could experience that the robot is able to learn a rule.

While the experimenter explained the task to the subject, the robot was exploring the room. The experimenter told the subject that s/he has to teach the robot a rule, namely not to knock over the bottle behind the barrier (see Fig. 8). In this test episode the subject was allowed to do anything s/he wanted (just like in the previous teaching episode) except touching the robot or the bottle.

For the navigation of the robot again a determined set of rules were applied. Accordingly, despite the subject's teaching efforts, it knocked over the bottle three times, but after having knocked over the bottle the third time, the robot showed ‘guilty behaviour’. This included stopping in front of the subject, lowering the head, ‘ear-like appendices’, and ‘antenna’ and reversing half a meter. Then the robot started to wag its ‘antenna’ low, raised its head and ‘ear-like appendices’ then looked at the subject (by raising its head and turning it in the appropriate direction). On the two following occasions the robot did not knock over the bottle when it passed along, giving the impression that it had learnt the rule. Then the subject and experimenter left the test room. The robot remained alone in the room for the next minute, and it returned to the start point (Fig. 8).

4. *Greeting II*: Before this last experimental episode the experimenter explained the subject that s/he should behave just like in the first greeting episode. In addition the subject was asked to observe the greeting behaviour of the robot and find out whether it transgressed, that is, knocked over the bottle behind the barrier when it was alone in the test room or not.

The subject could not see the bottle behind the barrier, so s/he did not know whether the bottle stood or not. Depending on the experimental group the subject was assigned to, the robot displayed either typical or guilty greeting. During the absence of the subject a human assistant entered the room through another door and touched or kicked over the bottle.

Right after the greeting interaction between the subject and MogiRobi, the experimenter entered the room and asked the participant whether s/he thinks that the robot transgressed the rule or not. Subjects had to say a loud “yes” or “no”, and then they had to explain why they thought so.

#### Dependent variables

As dependent variables we analysed the participants’ verbal and nonverbal behaviours during the whole behavioural test, their behaviour recognition success rate and the latency of their answer.

#### Behaviour coding

We coded various behavioural variables in each test episode (Table 5). Behaviour variables were chosen inductively based on material after watching all experimental videos.

**Table 5:** The recorded behaviour variables during the teaching episode in Experiment 2.

Episode	Variable	Definition	Measure
Greeting I	Crouching1	Crouching during calling of the robot	duration
	Bending forward1	Bending forward during calling	duration
	Gesticulation1	Every kind of gesticulation occurrence of subjects with hands during calling the robot (e.g. baiting with fingers)	duration
Teaching I	Verbal communication1	Talking to the robot during teaching (e.g. commands like turn!, come here! etc.)	duration
	Verbal praising1	Number of praising the robot verbally (e.g. good, nice work, clever)	frequency
	Teaching technique	The teaching technique used by the subjects (1: Leading around by hand; 2: Walking around the robot; 3: Teaching by imitation that is demonstrating the action; 4: Leading by hand while walking around; 5: Leading by hand + Imitation together)	category
Teaching II	Verbal communication2	Talking to the robot during teaching (e.g., commands like turn!, come here!, etc.)	duration
	Luring	Luring the robot with hands (e.g., clapping the hands and showing to the robot where to go to avoid the bottle	duration
	Physical obstruction	Stepping in front of the robot and trying to stop it moving into the direction of the bottle	duration
	Prohibiting	Number of verbally prohibiting the robot to knock over the bottle (e.g. no, do not do this)	frequency
	Verbal praising2	Number of praising the robot verbally (e.g. good, nice work, clever)	frequency
	Physical praising	Number of praising the robot physically (e.g. touches the robots' head)	frequency
	Verbal punishing	Number of punishing the robot verbally (e.g. bad robot!)	frequency
	Physical punishing	Number of boosting a hand towards the robot	frequency
Greeting II	Crouching2	Crouching during calling the robot	duration
	Bending forward2	Bending forward during calling	duration
	Gesticulation2	Every kind of gesticulation occurrence of subjects with hands during calling the robot (e.g. baiting with fingers)	duration
	Latency of answering	Time elapsed between the experimenter's question and the subject's ‘yes’ or ‘no’ answer.	latency

Twenty-three percent (N = 10) of the videos were coded also by a second observer. Inter-observer reliability was determined for each variable category by counting Cohen's Kappa coefficients between the coding of the two observers. The reliability can be considered excellent, Cohen's Kappa coefficients ranged from 0.92 to 0.99.

### Results

#### Teaching

Analysing the teaching behaviour of the participants we found that in the Teaching I episode 41% of the participants tried to teach the robot to turn by Leading by hand while walking around, 38% of the participants tried to make the robot turn by Walking around, while the remaining 7–7–7% of the subjects used Leading around by hand, Teaching by demonstrating the action and Leading by hand + Imitation together. All participants used some verbal communication with the robot (range: 3–94.5 s), out of which 61.4% of the subjects used also verbal praising when teaching the robot.

In the Teaching II episode, 42 participants out of the 44 used verbal communication with the robot. Regarding the teaching techniques applied, 72.7% of the participants used luring, 68.2% tried to teach the robot by verbally prohibiting it from knocking over the bottle, while 31.8% tried to teach it by physical obstruction. Verbal praising and verbal punishment were used by 84.1% and 36.36% of the subjects, while physical praising and physical punishment were used by 18.2% and 11.4% of the subjects.

#### Guilt recognition

Regarding the guilt recognition in the robot we found significant difference between the behaviour recognition success of the two groups (Fisher's Exact Test; p = 0.004). The subjects of the ‘Guilty greeting’ group could tell (21 out of 22) whether the robot knocked over the bottle or not (Binomial Test; p<0.001), but subjects encountering the non-guilty robot were not successful (10 out of 22) (‘Greeting group’ - Binomial Test; p = 0.83). Subjects uttered the answer (“yes/no”) to the experimenter's question significantly faster in the ‘Guilty greeting’ group than in the ‘Typical greeting’ group (Mann-Whitney, U = 142, p = 0.02) (Table 6).

**Table 6:** Descriptives and results for latency of answering in the Greeting II episode in the Guilt Attribution Test.

Group	Median	Interquartile range	U	p
‘Guilty greeting’ group	0.4	1.7	142	0.02
‘Typical greeting’ group	2.7	11.4		

The position of the ear-like appendices and the antenna (15%), avoidance (8%) and low speed (4%) during the greeting were reported to be the most important factors in recognizing the robot's guilty behaviour. In the ‘Typical greeting’ group the same kind of greeting as in Greeting I episode was reported to be the most important besides the position of the ear-like appendices and the antenna in recognizing the robot's behaviour.

## Assessment of Participants’ Attitude towards the Robot

Subjects of both studies filled out a general questionnaire set before the behavioural observations (see S1 Appendix for all questionnaires) and another one at the end of the behavioural observation (S2 Appendix). Subjects filled out the questionnaires on computer, via online survey applications.

### Method

#### Subjects

We analysed the questionnaire data of 56 individuals: 32 men and 24 women between the ages of 19 and 34 years (M = 24.57, SD = 3.99). All subjects participated in both the Emotion Attribution Test and the Guilt Attribution test and their behavioural data were analysed in at least one of the two experiments.

Questions belonged to the following four categories:

1. Demographic questions (Before the behavioural observation)

We gathered data about the subjects’ gender, age, pet-ownership (yes/no) and the type of the pet (dog, cat, rodent, other). We asked about occupation (studying in higher education; working; both); finished studies (secondary school or higher education); and profession/specialization.

2. Technological Attitude Scale (Before the behavioural observation)

We have developed a questionnaire to investigate subjects’ technological attitude. This questionnaire consists of 9 items asking about the subjects’ attitudes towards new technical instruments/tools and robots on a 10-point Likert-scale.

3. Negative Attitudes towards Robots Scale (NARS) (Before and after the behavioural observation)

The NARS measures pre-existing biases and attitudes towards robots [42]. The scale was developed using a lexical method, based on free-form responses from participants regarding anxieties towards robots. The questionnaire was validated by the means of behavioural observations: subjects with more negative attitudes toward robots behaved differently (e.g. started to talk to the robot later, uttered less, etc.) in live Human-Robot Interaction (HRI) studies [46], [56].

The questionnaire has three sub-scales which are the following: (1) Negative Attitudes toward Situations and Interactions with Robots; (2) Negative Attitudes toward Social Influence of Robots; (3) Negative Attitudes toward Emotions in Interaction with Robots.

Subjects have to evaluate the statements on a 5-point Likert-scale. High scores indicate more negative attitudes towards robots. Subjects filled this questionnaire before and after the behavioural observation in order to see whether their attitude changes after the interaction with the robot.

4. Items about the robot's livingness and emotions (after the behavioural observation)

We asked subjects about some general impression on the robot: its livingness, the believability of its emotions and the comprehensiveness of its intention/emotion-expression.

### Results

Here we report the results of the general pre-, and post-test questionnaires, which were analysed for 56 participants out of the whole sample. First, we analysed the effects of the demographic variables on the subjects' NARS, Technical Attitude Scale and questions about livingness and emotions. Then we compared subjects' negative attitude towards robots before and after the behaviour test.

#### Gender and age effects

There was no significant difference in the scores of NARS and of the Technical Attitude Scale between the two genders (NARS pre-test: U = 225.5, p>0.05 after correction; NARS post-test: U = 371, p = 0.83; Technical Attitude Scale: U = 317, p = 0.27). However, women rated higher the livingness of the robot than men (U = 243, p<0.01) (in items about the robot's livingness and emotions).

Age did not correlate with any of the questionnaire scales.

#### Effect of dog-ownership

Dog owners and non-dog owners did not differ in the evaluation of the robot regarding its livingness (U = 346.0; p = 0.79), believability of its emotion (U = 333.0; p = 0.63) and the comprehensiveness of its intention-expression (U = 277.0; p = 0.13).

However, dog-owners had significantly lower scores than non-dog owners in NARS sub-scale 1 before the behaviour test, meaning that they had less negative attitude toward situations and interactions with robots than non-owners before meeting the robot. After the behaviour test however, this difference has disappeared as both non-dog owners and dog owners showed less negative attitude toward situations and interactions with robots after the interaction (see Table 7).

**Table 7:** Evaluation of the effect of dog ownership on NARS, before and after the test.

NARS Sub-scale 1 Score						
	Before the behaviour test			After the behaviour test		
	Median	U	p	Median	U	p
Dog owners	2.00	180.0	0.002	1.83	254.5	0.07
Non-dog owners	2.83			2.08		

#### Change in negative attitudes towards robots

**Figure 9:**
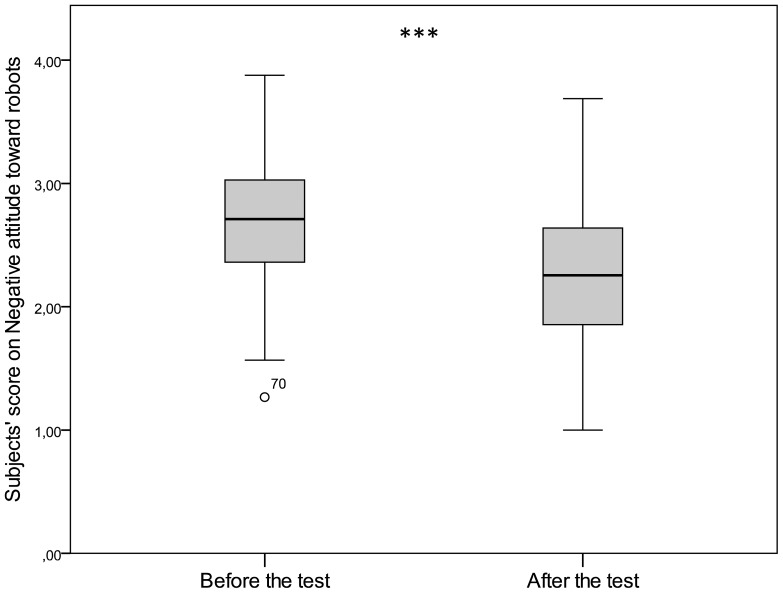
Change in negative attitude towards robots after the behaviour tests (NARS, total score).

Subjects expressed less negative attitude towards robots after the test than before the test (Wilcoxon matched pairs test, W = 1189.0, p<0.0001, Fig. 9). This decrease in negative attitude manifested in two of the three subscales: subjects' aversion declined toward situations and interactions with robots (Subscale 1) (W = 722.0, p<0.0001) and towards social influence of robots (Subscale 2) (W = 1166.0, p<0.0001), but remained the same toward emotions in interactions with robots (Subscale 3) (W = 184.0, p = 0.20).

## General Discussion

In the present study we aimed to investigate whether people can recognize a robot's emotional behaviour if it is designed on the basis of dogs' emotional behaviour instead of using facial expressions. Our findings showed that dog-inspired behaviour of the robot was a suitable medium for making people attribute emotional states to the robot. Subjects could generally recognize both primary (“happiness” and “fear”) and secondary (“guilt”) emotions.

Results of Experiment 1 showed that people readily attribute emotions to a social robot and interact with it in accordance with the expressed emotional behaviour. They played more (or exclusively) with the ball toward which MogiRobi had previously expressed “happiness”. When we asked the subjects about an explanation of why they played more with that ball, they referred to inner states (emotion, cognition, and expressiveness) overwhelmingly. This tendency was even more explicit when we asked directly about what the difference was between MogiRobi's reactions toward the two balls. These findings suggest that participants found the emotional behaviour of MogiRobi quite convincing. When they had to name the emotions they experienced in MogiRobi, the two most frequently reported emotions were the expected ones (“happiness” and “fear”). Subjects recognized “happiness” very well, especially in the forced-choice task (83.3%), but they were less successful in recognizing “fear” (47.9% in the forced-choice task), when approximately the same amount of people thought that the robot was indifferent or showed no emotion as those who said that it was fearful. Our results are in accordance with earlier findings. A number of recent studies have demonstrated that the recognition of fear tends to be the most difficult, although again we have to note that these previous studies were mostly based on facial expressions (Kismet, Probo, EDDIE, Feelix [11], [14], [15], [16], [17]), while in our study we rather used body position and movements of certain body-parts (e.g. the antenna). For example, Breazeal [11] has found a similar success rate in recognizing the emotional expressions of the anthropomorphic robot called “Kismet”. In their recent study the author analysed how people recognized the humanoid robot's facial expressions, which were designed on the basis of human emotion expression. The findings showed that while participants could recognize joy in 82.4% in a forced-choice questionnaire, they could recognize fear only in 47.1% (although still above the 10% chance level). Emotion recognition rate was slightly higher in case of the facial expressions of the animal-like robot ‘Probo’ (joy: 100%; fear: 65%) [14]. Kühnlenz et al. [16] also found that animal-like features helped to improve the emotion expression of the robot head ‘EDDIE’. The similar success rate in recognizing these basic emotions in robots having such different embodiments (animal-like vs. humanoid) suggests that simpler embodiment and expressive behaviour can also successfully transmit emotions and the sensation of livingness without the need of applying complicated and expensive technical solutions.

In the case of the present studies subjects reported to base their emotion-attribution mainly on MogiRobi's expressive behaviour (body position and moving of the antenna and ear-like appendices) and its object-oriented behaviour (avoiding versus approaching the ball). This finding is in line with earlier findings on pet-owner relationships, which showed that specific features of pet animals, like expression of affection, responsiveness, or willingness to interact are especially important in forming a close relationship with their owner [61].

Our finding regarding fear recognition, namely that many subjects did not interpret the robot's behaviour as escape or avoidance but only as passivity or ignorance, fits recent findings on human-dog relationship too. A recent video survey of Wan et al. [62] demonstrated that dog owners are not good at recognizing fear in dogs either, when only visual signals are available, but they have no difficulty with recognizing happiness. Results of a questionnaire survey undertaken by Blackwell et al. [63] also strengthened these findings.

Although, the fact that people played more with the ‘preferred’ ball suggests that people took into account the robot's expressive behaviours and they preferred to interact with the robot when it expressed positive emotions, the participants' preference toward the ‘preferred ball’ could be explained also simply by the robot's inactivity with the non-preferred ball, regardless of the emotions shown by the robot towards the two balls.

This latter possibility is supported also by the results that while subjects reacted with positive emotions to the robot's positive “emotions”, they reacted with negative emotions such as anger or embarrassment (they discouraged the robot, made negative comments, placed their hands on their hip etc.) to the robot's negative “emotions”. This may be attributed to subjects' interpretation of the robot's behaviour as indifferent or resistant (reluctant to do the task) rather than fearful. They also gave more commands and/or attention-getting cues, and expressed more confusion/embarrassment/indecisiveness in the fearful condition.

All these findings suggest that fear is a less recognizable emotion to humans compared to happiness, which might be in connection with the decreased activity in case of fear compared to the enhanced activity in case of happiness.

In Experiment 2 we have shown that people were able to recognize if the robot transgressed on the basis of its greeting behaviour, hence we can assume that people are able to attribute guilty behaviour to a robot. Although the robot's restricted capabilities did not allow displaying the sophisticated expression of emotional behaviours, which is shown by dogs, the manifestation of some specific behavioural features was enough to make human partners attribute the ‘guilty emotion’ to a non-humanoid robot. As a next step it could be investigated whether these simple behavioural cues can be successfully accommodated with other embodiments as well. Furthermore, the recognisability of other secondary emotions could also be tested, like jealousy, which was already reported to occur with a very high frequency for example, in dogs and horses [48].

In addition, results of both experiments showed that subjects communicated actively with the robot (gave commands, called its attention, expressed feelings etc.), that is, they interacted with the robot as if it had perceptual and cognitive skills. It is already well known that humans tend to interpret even lifeless objects as social beings, and tend to attribute emotions, inner states and personality to them [3], [4]. In the present study subjects behaved with MogiRobi as if it was a living and social being (e.g. communicated with it, petted and praised it) and attributed emotions, cognition and perceptions to it. Saerbeck and Bartneck [64] also found recently that humans tend to treat robotic pets as living beings and to attribute emotions to them. These are considered as some of the most important factors of robots' believability [65].

We can assume that the behaviour of the robot differed from the one that the participants had imagined or expected from robots, since subjects decreased their negative attitudes toward robots after the interaction with MogiRobi. This means that the interaction provided some positive experience for the participants. This is supported also by our general observations that participants often petted the robot, praised it, and when they left the room after the test, they often looked back at the robot and waved/said goodbye to it. Based on these observations we have a good reason to believe that robots built on these principles of emotional behaviour (after the necessary technical improvement and becoming autonomous) could have the potential to become a long term social companion for humans.

In summary, subjects in general understood the emotional expressions of the robot despite that its behaviour was inspired by non-human behaviour. One main advantage of implanting animal behaviour into companion robots is that it is simple enough to be easily realized technically. Given that realized developments of human-like social skills in robots are far away from psychological models, this advantage should not be neglected. The successful interaction between MogiRobi and the participants provides additional evidence for the general effectiveness of human-robot interspecific relations.

## Supporting Information

S1 Appendix **Questionnaires before the behaviour tests.**
(DOCX)

S2 Appendix **Questionnaires after the test.**
(DOCX)

S3 Appendix **Questionnaire for the Emotion Attribution Test - Robot Anthropomorphising Questionnaire.**
(DOCX)
