# Correction: Emotion Attribution to a Non-Humanoid Robot in Different Social Situations

**DOI:** 10.1371/journal.pone.0121929

**Published:** 2015-03-25

**Authors:** The PLOS ONE Staff

There is an error in affiliation 1 for authors Gabriella Lakatos, Márta Gácsi, and Boróka Bereczky. Affiliation 1 should be: MTA-ELTE Comparative Ethology Research Group, Pázmány Péter sétány 1/C, Budapest 1117, Hungary.
