# Supplementary material for: Emotion Attribution to a Non-Humanoid Robot in Different Social Situations
Source: PLoS One. 2014 Dec 31;9(12):e114207. doi: 10.1371/journal.pone.0114207 (PMC4281080; doi:10.1371/journal.pone.0114207)
Supplement: S1 Appendix — Questionnaires before the behaviour tests. (DOCX) [file pone.0114207.s001.docx]

**Appendix S1**

Questionnaires before the behaviour tests

*Demographics*

Sex:

Age:

Profession:

If you are a student, what do you study? ................................................

Highest level of education:

1.) primary school

2.) vocational school, -please specify the profession as well: .......................................

3.) high school graduation

4.) college/ university, -please specify the university and the department as well: .................................................

Do you have a pet? No / Yes, namely: ..............................................................

Do you have a robot? No / Yes, namely: .....................................................

*Technological Attitude Scale*

| I enjoy exploring new technical devices (1: not true at all - 10: absolutely true) | 1 – 2 – 3 – 4 – 5 –  6 – 7 – 8 – 9 – 10 |
| --- | --- |
| My technological knowledge is excellent (1: I strongly disagree – I absolutely agree) | 1 – 2 – 3 – 4 – 5 –  6 – 7 – 8 – 9 – 10 |
| I can imagine to have a „companion” robot at home, which I can cooperate agreeably with (it greets me, chats, etc.) | 1 – 2 – 3 – 4 – 5 –  6 – 7 – 8 – 9 – 10 |
| I would enjoy if a robot made the housework instead of me. | 1 – 2 – 3 – 4 – 5 –  6 – 7 – 8 – 9 – 10 |
| I would enjoy if a robot took a lot of work from me at my workplace. | 1 – 2 – 3 – 4 – 5 –  6 – 7 – 8 – 9 – 10 |
| I would enjoy if I had a „companion robot” who I had to care for. | 1 – 2 – 3 – 4 – 5 –  6 – 7 – 8 – 9 – 10 |
| I’m afraid that robots will be used for bad purposes in the future. | 1 – 2 – 3 – 4 – 5 –  6 – 7 – 8 – 9 – 10 |
| I would gladly buy a „companion robot” for myself. | 1 – 2 – 3 – 4 – 5 –  6 – 7 – 8 – 9 – 10 |
| Generally I think that robots are useful. | 1 – 2 – 3 – 4 – 5 –  6 – 7 – 8 – 9 – 10 |

*Negative Attitudes towards Robots Scale*

Rate the following statements on a five-point scale according to how much you think they are correct.

The number of grades for each item is five (1: I strongly disagree, 2: I disagree, 3: Undecided, 4: I agree, 5: I strongly agree).

1. I would feel uneasy if robots really had emotions.

(I strongly disagree) 1---2---3---4---5 (I strongly agree)

2. Something bad might happen if robots developed into living

beings.

(I strongly disagree) 1---2---3---4---5 (I strongly agree)

3. I would feel relaxed talking with robots.

(I strongly disagree) 1---2---3---4---5 (I strongly agree)

4. I would feel uneasy if I was given a job where I had to use robots.

(I strongly disagree) 1---2---3---4---5 (I strongly agree)

5. If robots had emotions, I would be able to make friends with them.

(I strongly disagree) 1---2---3---4---5 (I strongly agree)

6. I feel comforted being with robots that have emotions.

(I strongly disagree) 1---2---3---4---5 (I strongly agree)

7. The word “robot” means nothing to me.

(I strongly disagree) 1---2---3---4---5 (I strongly agree)

8. I would feel nervous operating a robot in front of other people.

(I strongly disagree) 1---2---3---4---5 (I strongly agree)

9. I would hate the idea that robots or artificial intelligences were

making judgments about things.

(I strongly disagree) 1---2---3---4---5 (I strongly agree)

10. I would feel very nervous just standing in front of a robot.

(I strongly disagree) 1---2---3---4---5 (I strongly agree)

11. I feel that if I depend on robots too much, something bad might

happen.

(I strongly disagree) 1---2---3---4---5 (I strongly agree)

12. I would feel paranoid talking with a robot.

(I strongly disagree) 1---2---3---4---5 (I strongly agree)

13. I am concerned that robots would be a bad influence on children.

(I strongly disagree) 1---2---3---4---5 (I strongly agree)

14. I feel that in the future society will be dominated by robots.

(I strongly disagree) 1---2---3---4---5 (I strongly agree)
