# Supplementary material for: Emotion Attribution to a Non-Humanoid Robot in Different Social Situations
Source: PLoS One. 2014 Dec 31;9(12):e114207. doi: 10.1371/journal.pone.0114207 (PMC4281080; doi:10.1371/journal.pone.0114207)
Supplement: S2 Appendix — Questionnaires after the test. (DOCX) [file pone.0114207.s002.docx]

**Appendix S2**

Questionnaires after the test

*Negative Attitudes towards Robots Scale*

(see in Appendix 2)

*Items about the robot’s livingness and emotions*

Do you think that the robot in the previous experiments expressed its intentions/ emotions comprehensible?

(not at all) 1---2---3---4---5 (totally)

How much do you think MogiRobi resembles to a living creature?

(not at all) 1---2---3---4---5 (totally)

How much did MogiRobi seem to have emotions?

(not at all) 1---2---3---4---5 (totally)
