# Supplementary material for: Emotion Attribution to a Non-Humanoid Robot in Different Social Situations
Source: PLoS One. 2014 Dec 31;9(12):e114207. doi: 10.1371/journal.pone.0114207 (PMC4281080; doi:10.1371/journal.pone.0114207)
Supplement: S3 Appendix — Questionnaire for the Emotion Attribution Test - Robot Anthropomorphising Questionnaire. (DOCX) [file pone.0114207.s003.docx]

**Appendix S3**

*Questionnaire for the Emotion Attribution Test - Robot Anthropomorphising Questionnaire*

1. Which ball did you play more with MogiRobi in the “free play” phase? (underline it)

*Black-and-white one / Yellow one*

1. Why did you play more with *that* ball?

…………………………………………………………………………………………

………………………………………………………………………………………….

1. Did you experience that MogiRobi reacted differently to the two different balls? *Y/ N*

1. What was the difference?

…………………………………………………………………………………………..

1. How much do you think MogiRobi resembles to a living creature?

(not at all) 1---2---3---4---5 (totally)

1. How much do you think MogiRobi possesses (or seems to possess) emotions?

(not at all) 1---2---3---4---5 (totally)

1. What kind of emotions have you experienced in MogiRobi?

…………………………………………………………………………………………………

1. What kind of emotion did MogiRobi show toward the *yellow* ball?

…………………….......………………………

1. What kind of emotion did MogiRobi show toward the *black-and-white* ball?

…………………………………………………

1. What kind of emotions did MogiRobi show toward the *yellow* ball from the following? (forced choice)

- joy
- sadness
- fear
- anger
- surprise
- disgust
- no emotion
- other: _____________________

1. What kind of behavior elements of the robot do you base your response on? (What did the robot do that made you think that it had the given emotion?)

………………………………………………………………………………………………

1. What kind of emotions did MogiRobi show toward the *black-and-white* ball from the following? (forced choice)

- joy
- sadness
- fear
- anger
- surprise
- disgust
- no emotion
- other: _____________________

1. What kind of behavior elements of the robot do you base your response on? (What did the robot do that made you think that it had the given emotion?)

…………………………………………………………………………………………………..
